# The Candidate Antimalarial Drug MMV665909 Causes Oxygen-Dependent mRNA Mistranslation and Synergizes with Quinoline-Derived Antimalarials

**DOI:** 10.1128/AAC.00459-17

**Published:** 2017-08-24

**Authors:** Cindy Vallières, Simon V. Avery

**Affiliations:** School of Life Sciences, University of Nottingham, University Park, Nottingham, United Kingdom

**Keywords:** translation fidelity, iron-sulfur cluster, oxidative stress, Medicines for Malaria Venture, malaria, antimalarial, yeast

## Abstract

To cope with growing resistance to current antimalarials, new drugs with novel modes of action are urgently needed. Molecules targeting protein synthesis appear to be promising candidates. We identified a compound (MMV665909) from the Medicines for Malaria Venture (MMV) Malaria Box of candidate antimalarials that could produce synergistic growth inhibition with the aminoglycoside antibiotic paromomycin, suggesting a possible action of the compound in mRNA mistranslation. This mechanism of action was substantiated with a Saccharomyces cerevisiae model using available reporters of mistranslation and other genetic tools. Mistranslation induced by MMV665909 was oxygen dependent, suggesting a role for reactive oxygen species (ROS). Overexpression of Rli1 (a ROS-sensitive, conserved FeS protein essential in mRNA translation) rescued inhibition by MMV665909, consistent with the drug's action on translation fidelity being mediated through Rli1. The MMV drug also synergized with major quinoline-derived antimalarials which can perturb amino acid availability or promote ROS stress: chloroquine, amodiaquine, and primaquine. The data collectively suggest translation fidelity as a novel target of antimalarial action and support MMV665909 as a promising drug candidate.

## INTRODUCTION

The malaria parasite, Plasmodium, is a major public health burden in the developing world. More than 200 million new cases of malaria were reported globally in 2015, with Plasmodium spp. responsible for 438,000 deaths that year, mainly of children and pregnant women in sub-Saharan Africa (1). Despite the availability of antimalarial drugs for treatment, there is an urgent need for novel inhibitors as the parasite develops resistance to first-line therapies, compromising the treatment of malaria patients. To support current therapy and help eradicate malaria, new drugs with novel modes of action and no cross-resistance with current antimalarials are necessary (2).

A common strategy for identifying new potential drugs is to screen *in vitro* cultures of Plasmodium spp. against large chemical libraries, typically assaying for growth inhibition. Determining the molecular target(s) of candidate agents identified from such screens is challenging. One approach relies on the generation of resistant strains by prolonged exposure of parasites to drugs and identification of resistance-associated mutations by whole-genome sequencing (3, 4). However, identification of generic resistance mechanisms shared by diverse compounds is common. For example, several groups of investigators have identified resistance mutations in Plasmodium falciparum ATP4 (PfATP4), a Na^+^/H^+^-ATPase regulating parasite Na^+^, after exposure of Plasmodium spp. to diverse ranges of new agents (5–7). It is still unclear why PfATP4 appears to be a resistance marker for so many recently discovered drugs although other transporters such as the P. falciparum chloroquine (CQ) resistance transporter and multidrug resistance gene 1 (PfCRT and PfMDR1, respectively) have also been associated with resistance to current antimalarials that do not necessarily have common mechanisms of action (8). An effective strategy for specifying mode of action can be to screen for compounds targeting a specific function, typically involving a transgenic-parasite assay. Unfortunately, despite recent improvements (e.g., genome editing with CRISPR/Cas9 [9]), the main human malaria parasite P. falciparum is not easy to manipulate genetically. Among alternative experimental systems, the yeast Saccharomyces cerevisiae is a powerful eukaryotic model for mode-of-action studies as it is inexpensive to culture and easy to manipulate and offers an extensive range of genetic tools and libraries (10). With strong conservation of function between yeast and Plasmodium spp., yeast has been widely used for heterologous expression of functional Plasmodium sp. proteins (11–14) and for studies elucidating antimalarial drug modes of action (15–19) or resistance (20–22). Findings from such yeast studies have been successfully extrapolated to malaria patients (23).

Protein synthesis, as an essential function of the cell, represents an attractive drug target. Plasmodium spp. possess three genomes: nuclear, apicoplastic (from a relic chloroplast), and mitochondrial. All three genomes require dedicated translational machineries to function (24). Antibiotics targeting organellar components required for protein translation, specifically organellar ribosomes and tRNA synthetases, have long been used to help treat and prevent infections by the parasite (25). Recently, a potent new drug, DDD107498, has been reported to inhibit protein synthesis of P. falciparum at multiple life cycle stages through eukaryotic translation elongation factor 2 (eEF2) which is necessary for GTP-dependent ribosome translocation along mRNA (3). To date, no antimalarials have been described that target the fidelity of protein synthesis. Antibiotics such as aminoglycosides that act via mRNA mistranslation have proven very effective against bacteria (26).

The Medicines for Malaria Venture (MMV) distilled over 25,000 compounds that kill blood stages of P. falciparum in vitro into a group of 400 chemically diverse compounds with minimal cytotoxicity, called the Malaria Box (27, 28). In the present work, we tested a number of these compounds in combination with the aminoglycoside antibiotic paromomycin in order to identify agents potentially targeting protein synthesis. Previously, synergistic inhibition of yeast growth in combination with paromomycin led to characterization of a novel mode of action of the toxic metal chromate, based on errors in mRNA translation during protein synthesis (29). Here, we reveal one compound [MMV665909; 2-bromo-*N*-(4-pyridin-2-yl-1,3-thiazol-2-yl)benzamide] among the MMV drugs tested that produces synergistic growth inhibition with paromomycin. Using reporters of mistranslation and other yeast genetic tools not available with the malaria parasite, we corroborate a role of the MMV compound in mistranslation. Promisingly, we also observed synergy between MMV665909 and three existing antimalarials: amodiaquine, chloroquine, and primaquine. The results suggest a novel target for a candidate antimalarial, which additionally exhibits synergy when combined with quinoline derivatives.

## RESULTS

### Discovery of a novel antimalarial drug candidate targeting protein translation.

**FIG 1:**
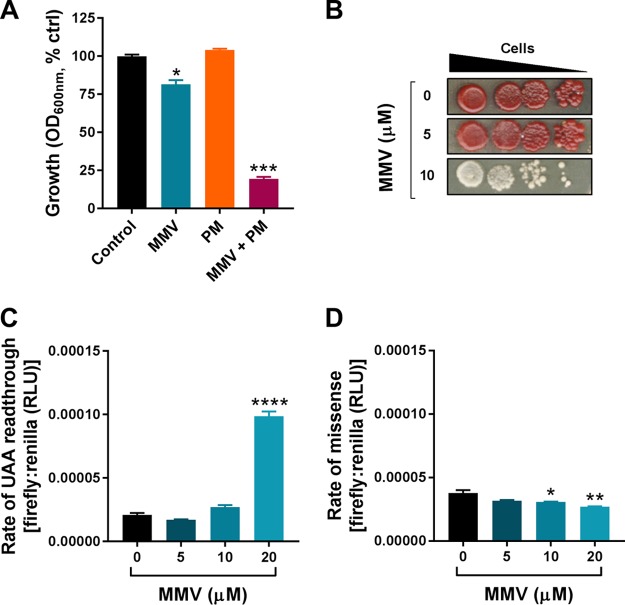
MMV665909 acts synergistically with paromomycin and causes stop codon readthrough. (A) Growth of S. cerevisiae in YPD broth alone or supplemented with 25 μM MMV665909 (MMV) and/or 200 μg · ml^−1^ paromomycin (PM). The OD_600_ was measured after 15 h. Growth was calculated as a percentage of growth of the control (ctrl) without drug. (B) A tenfold dilution series of S. cerevisiae W303 (*ade2-1*) was spotted from left to right on YPD agar alone or supplemented with MMV665909. Loss of red pigmentation indicates readthrough of the premature stop codon associated with the *ade2-1* allele. (C) S. cerevisiae transformed with the dual-luciferase plasmid containing a UAA stop codon between the firefly and Renilla luciferase ORFs was exposed to the indicated MMV665909 concentrations in YPD agar before determination of both luciferase activities. The ratio of these activities indicates the level of translation readthrough at the UAA stop codon. (D) S. cerevisiae transformed with the dual-luciferase plasmid containing a His245 → Arg245 missense codon within the firefly luciferase ORF was assayed as described for panel C. The firefly/Renilla luciferase ratio here provided a measure of amino acid misincorporation. Mean data are shown in panels A, C, and D from triplicate independent experiments ± standard errors of the means. *, *P* < 0.05; **, *P* < 0.01; ***, *P* < 0.001; ****, *P* < 0.0001, by two-tailed Student's *t* test. RLU, relative light units.

In a recent study, the 400 compounds comprising the Malaria Box were screened for growth inhibition of the yeast model Saccharomyces cerevisiae. At the highest drug concentration supplied (50 μM), only 16 of the drugs produced detectable growth inhibition (28). To help identify compounds that may perturb protein translation in the present study, we tested for synergy with the aminoglycoside paromomycin. Paromomycin is known to cause mRNA mistranslation via ribosome binding (26, 30) and has been applied successfully previously to discover mistranslation-based action of other agents (29). Synergy is evident where a growth effect is significantly stronger with combined drugs than from simple addition of their individual effects, indicating that the compounds target a common process (31). Each drug was supplied at just subinhibitory concentrations, and growth inhibition was calculated after 15 h. Four of the MMV drugs most active against yeast were tested for synergy (Fig. 1A; see also Fig. S1 in the supplemental material). Among these, MMV665909 produced a significant, ∼80%, inhibition of yeast growth when combined with the aminoglycoside (Fig. 1A). Synergy was quantified by calculation of a combination index (CI) using a response additivity approach (32). The CI for the MMV665909-paromomycin combination was 0.23, indicating that the drugs act synergistically (a CI of <1 is considered indicative of synergy).

The above evidence for synergy suggested that MMV665909 and paromomycin may target a common process. As paromomycin causes mistranslation, we tested whether MMV665909 also causes mRNA mistranslation, in the first instance using a qualitative yeast assay based on readthrough of a premature *ade2-1* UAA stop codon. Mistranslation-dependent readthrough suppresses the red pigmentation associated with this allele (29). MMV665909 suppressed the red pigmentation at a drug concentration which only slightly inhibited yeast growth (Fig. 1B). To support this qualitative indication of mistranslation, the rate of translational readthrough of a UAA stop codon was monitored quantitatively in a dual-luciferase assay. The plasmid used for this encodes two luciferases, Renilla followed by firefly, separated by the UAA stop codon. Expression of the firefly luciferase occurs when there is readthrough of the stop codon. The rate of readthrough was increased ∼5-fold in the presence of the MMV drug (Fig. 1C). In addition, decreased accuracy of translation elongation in the presence of MMV665909 was tested using a modified firefly luciferase construct containing a near-cognate His245 → Arg245 mutation, where misincorporation of histidine is required to restore wild-type activity. In this case, the drug did not increase firefly luciferase activity (i.e., amino acid misincorporation) and actually produced a slight decrease (Fig. 1D). The results suggested that MMV665909 does not impair translation fidelity generally but has some specificity for translation termination.

### MMV665909 impairs growth and translation fidelity in an oxygen-dependent manner.

**FIG 2:**
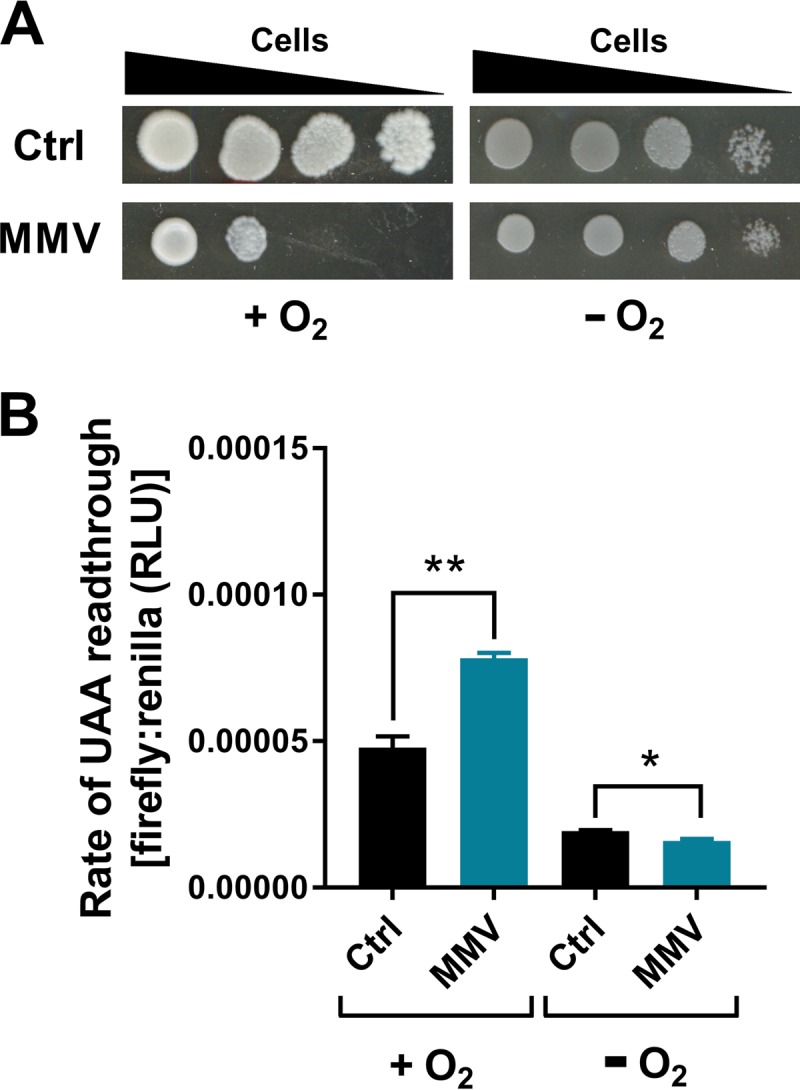
MMV665909 impairs growth and translation fidelity in an oxygen-dependent manner. (A) S. cerevisiae BY4741 in a 10-fold dilution series was spotted onto YPD agar alone or supplemented with 50 μM MMV665909 and incubated for 4 days under aerobic or anaerobic conditions. (B) Cells transformed with the UAA stop codon dual-luciferase plasmid were incubated in YPD broth with or without 20 μM MMV665909 and in the presence or absence of oxygen before luciferase activities were measured. Mean data are shown from triplicate independent experiments ± standard errors of the means. *, *P* < 0.05; **, *P* < 0.01, by two-tailed Student's *t* test.

Previous work showed that the metal toxicant chromate provokes protein synthesis defects via mRNA mistranslation (29); like MMV665909, chromate exhibited synergistic toxicity with paromomycin and increased the rate of stop codon readthrough. The chromate phenotype was oxygen dependent. To test whether the effect of MMV665909 on yeast growth was oxygen dependent, growth in the presence of drug was compared under anaerobic and aerobic conditions. Growth inhibition by MMV665909 was fully rescued in the absence of oxygen (Fig. 2A). The translation error rate was also compared using a dual-luciferase assay. The drug-induced stop codon readthrough observed under aerobic incubations was absent under the anaerobic condition (Fig. 2B). This indicates an oxidative basis for MMV665909-induced mistranslation. The background rate of mistranslation (in the absence of drug) was also decreased by the absence of oxygen. Chromate can cause mistranslation by competing with sulfate for uptake to cells via the Sul1 and Sul2 transporters, leading to cysteine and methionine starvation (33). In contrast, deletion of *SUL1* and *SUL2* did not rescue growth of yeast treated with MMV665909 (Fig. S2), thus distinguishing the action of MMV665909 from that of chromate. (An apparent slight sensitization of the *sul1Δ sul2Δ* deletion strain was not significant compared with growth of the corresponding wild-type controls.)

### Targeting of translation fidelity by MMV665909: involvement of the conserved iron-sulfur protein Rli1.

**FIG 3:**
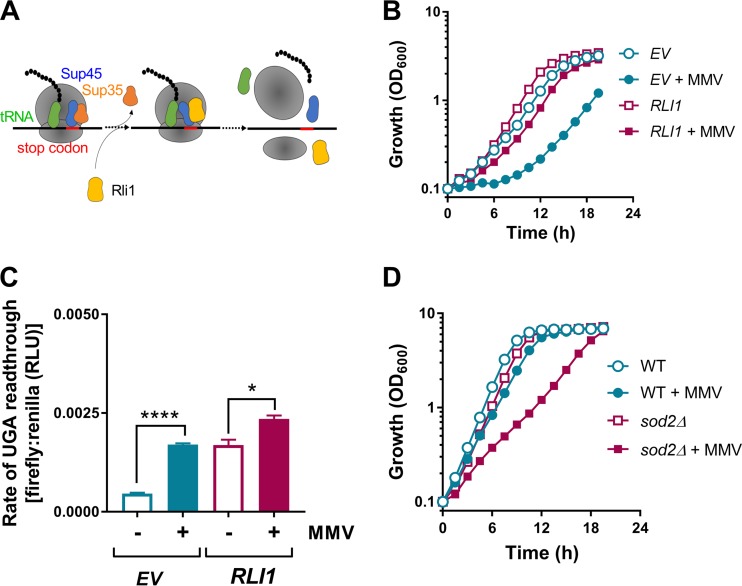
Involvement of Rli1 in MMV665909 action. (A) Simplified scheme showing the translation termination process. Rli1 and Sup45 are required for ribosome dissociation and termination fidelity. (B) S. cerevisiae cells transformed with a *tet*-bearing plasmid expressing an empty vector (EV) or overexpressing *RLI1* were cultured in YNB medium alone or supplemented with 10 μM MMV665909. Doxycycline was excluded to give maximal *RLI1* expression. Standard errors of the means from triplicate independent growth experiments are smaller than the dimensions of the symbols. (C) Yeast cells carrying the *tet*-bearing plasmid (empty vector or *RLI1*) and the dual-luciferase plasmid carrying a UGA stop codon (in a *BSC4* context) were incubated in the presence (+) or absence (−) of 20 μM drug, and luciferase activities were measured as described in Materials and Methods. Mean data are shown from triplicate independent experiments ± standard errors of the means. *, *P* < 0.05; ****, *P* < 0.0001, by two-tailed Student's *t* test. (D) Wild-type (WT) and isogenic deletion mutant *sod2*Δ strains were cultured in YPD medium alone or supplemented with 10 μM MMV665909 (in this experiment, unlike the experiment described in panel B, YNB medium was not needed for plasmid selection). Standard errors of the means from duplicate independent growth experiments are smaller than the dimensions of the symbols.

We showed above that MMV665909 impairs translation termination in an oxygen-dependent manner. Translation termination normally occurs when a stop codon enters the ribosomal A site during mRNA reading. Among the essential proteins involved in translation termination, function of the iron-sulfur (FeS) protein Rli1 (ABCE1 in human and other organisms) is known to be oxygen sensitive. Rli1 function is an important target of reactive oxygen species (ROS) and ROS-generating chemicals (34), including the antimalarial primaquine (PMQ) (18). In translation termination, Rli1 in concert with Sup45 (eukaryotic release factor 1 [eRF1]), dissociates and splits the ribosome into its subunits (35) (Fig. 3A), with Sup45 and Rli1 required for faithful stop codon reading (36–38). To indicate whether Rli1 may be targeted by MMV665909, we tested drug sensitivity in cells overexpressing the protein under *tet* control (34); increased expression of a principal drug target(s) should confer resistance to the relevant drug (39). Overexpression of *RLI1* conferred resistance to MMV665909 (Fig. 3B). Overexpression of *SUP45* produced a mild rescue in the lag phase but a mild sensitization in the exponential phase, with a net outcome of no effect after ∼20 h (Fig. S3). Overexpression of *RLI1* also appeared to moderate the extent of drug-induced stop codon readthrough, from 3.7-fold in the wild type to 1.4-fold in Rli1-overexpressing cells (Fig. 3C). These relative effects were unlikely to reflect saturation of the system (noting that the no-drug background rate was also increased in *RLI1*-overexpressing cells) as we have observed mistranslation rates of ∼0.004 with this construct. The increased background mistranslation rate of *RLI1*-overexpressing cells did not exert a marked growth effect and is possibly due to an imbalance in the translation machinery. Decreased Rli1 activity also is known to increase the rate of stop codon readthrough (37, 38). The data are consistent with the suggestion that Rli1 may be targeted by MMV665909. The dependency of Rli1 function on FeS biogenesis, which is rooted in the mitochondria, makes Rli1 ROS sensitive (34). The Mn superoxide dismutase Sod2 protects mitochondrial FeS clusters from superoxide attack, and we found that *sod2Δ* cells are hypersensitive to the MMV drug (Fig. 3D). The data support an oxidative mode of MMV665909 action on Rli1 function, an action that could account for mistranslation and growth inhibition.

### MMV665909 combined with quinoline derivatives produces synergistic inhibition of yeast growth.

**FIG 4:**
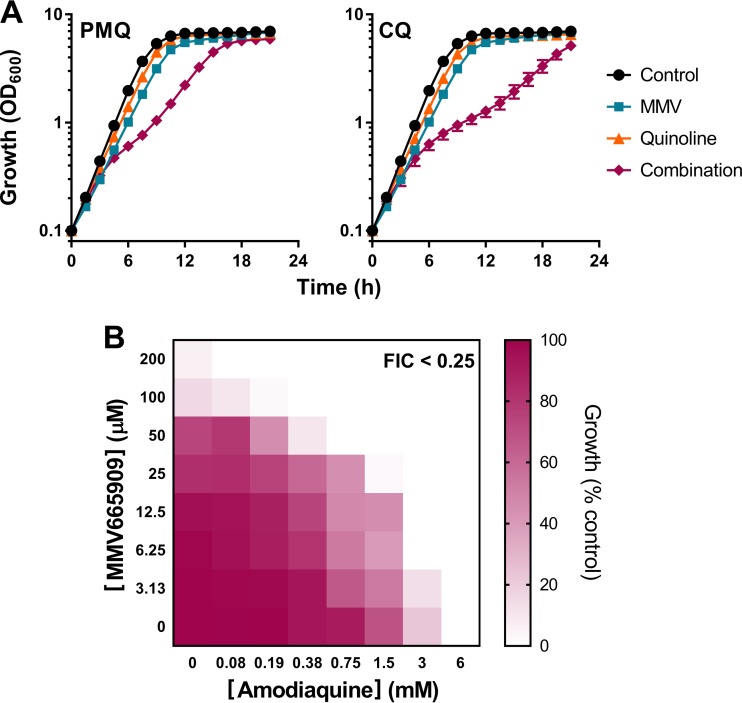
MMV665909 acts in synergy with current quinoline antimalarial drugs. (A) S. cerevisiae was cultured in YPD broth supplemented with 10 μM MMV665909, 1.5 mM primaquine (PMQ), and/or 5 mM chloroquine (CQ). Standard errors of the means from triplicate independent growth experiments are smaller than the dimensions of the symbols. (B) Checkerboard assay in YPD broth with yeast at the indicated concentrations of MMV665909 and amodiaquine. The growth values are percentages of the growth of the control (OD_600_) determined in the absence of both agents.

To decrease the likelihood of resistance emergence to antimalarials, the drugs are commonly used in combinations. We hypothesized that MMV665909 may act synergistically with certain quinoline-derived antimalarials as these also are known to cause oxidative stress as well as amino acid starvation (16), a potential cause of mRNA mistranslation (40, 41). To test the efficacy of MMV665909 in combination with the quinoline-containing antimalarials chloroquine (CQ), amodiaquine (AQ), and primaquine (PMQ), drugs were supplied at concentrations which, individually, were just subinhibitory. When combined, MMV665909 plus CQ and MMV665909 plus PMQ produced synergistic inhibition of exponential yeast growth (Fig. 4A). Amodiaquine could not be tested in the same way because of a drug color change during growth which produced a fluctuating contribution to optical density (OD) measurements. Therefore, AQ was tested in a checkerboard assay specifically for synergy. This showed that the combination of AQ with MMV665909 decreased the MICs of the individual agents by ≥8-fold and was synergistic, with a fractional inhibitory concentration (FIC) of 0.25 (combinations are considered synergistic when the FIC is <0.5) (Fig. 4B). The results indicated that MMV665909 produces synergistic growth inhibition when combined with currently used quinoline antimalarials, consistent with certain predicted overlaps in the actions of these drugs.

## DISCUSSION

With increased recrudescence of Plasmodium isolates resistant to current antimalarials, there is an urgent need for new drugs with broad therapeutic potential and new mechanisms of action to fight against malaria. One recent example is DDD107498, a novel multiple-stage antimalarial compound with clinical potential noted to target translation elongation factor 2, which is essential for protein synthesis (3). Due to its essentiality at all stages of the parasite life cycle, protein synthesis could be an important antimalarial drug target. Synergistic drug combinations that target specifically the fidelity of protein synthesis (in fungi) have been described previously (31). These combinations consisted of an aminoglycoside antibiotic and a sulfate transport inhibitor and produced synergistic inhibition against target organisms but not mammalian cells. Building on those findings, here we combined the aminoglycoside paromomycin (known to cause mRNA mistranslation) with compounds from the Malaria Box (27), testing for synergistic effects of the combinations using the yeast model. Synergy between drugs is commonly seen where they target a common process but by different mechanisms or pathways (31, 42), which is the principle applied here to find Malaria Box candidates that may target protein synthesis fidelity.

Many of the 400 diverse drug-like molecules in the Malaria Box do not affect yeast growth when tested individually. One reason for this high level of resistance is attributable to expression by yeast of efficient drug efflux pumps (28). Elsewhere, the antimalarial drug atovaquone is known to inhibit complex III of the yeast mitochondrial respiratory chain *in vitro* (43) but does not inhibit growth due to efficient drug efflux (44). Another contributory factor to resistance against certain MMV drugs could be where these target respiration (28) since yeast is commonly cultured under fermentative conditions, as was the case here where we were not concerned with respiratory drug targets. Moreover, one compound, MMV665909, acted in synergy with paromomycin in this study. MMV665909 also provoked stop codon readthrough. MMV665909 was not identified as a translation inhibitor of P. falciparum in a previous high-throughput *in vitro* translation screen of the Malaria Box (45). However, these investigators did not test mistranslation. Rather, their assay specifically probed the level of protein (luciferase) synthesis in an *in vitro* translation system. The luciferase assay used in this study is based on a dual-luciferase system designed to assay specifically for mistranslation, according to relative expression levels of two luciferases (46, 47). Therefore, results from the two studies are not inconsistent, and this work highlights the importance of using different assay formats for deep interrogation of agents that may impair protein synthesis at different levels or by different mechanisms.

MMV665909 caused mRNA mistranslation in an oxygen-dependent manner. Consistent with an oxidation-related mechanism, MMV665909 scored fourth highest among all the Malaria Box compounds for predicted propensity to form highly reactive epoxides during metabolism (28). Elsewhere, oxygen-dependent chromate-induced mistranslation is known to lead to an accumulation of toxic protein aggregates and loss of cell viability (29). Chromate interferes with mRNA translation indirectly by competing with sulfate for uptake into cells, leading to starvation for the sulfur-containing amino acids (cysteine and methionine) needed for protein synthesis (33). Unlike the effect of chromate, deletion of the relevant sulfate transporters did not alter MMV665909 resistance, indicating a different mechanism. As the effect of MMV665909 appears to be specific to the termination of translation (no rescue of the firefly luciferase activity was observed when a missense mistranslation was assayed), we investigated rescue by Rli1 as Rli1 is required for translation termination but not translation elongation (48) and is known to be ROS sensitive (34). Overexpression of Rli1 conferred MMV665909 resistance and also partly rescued the effect of the MMV drug on mRNA mistranslation. Rli1 is a highly conserved (49, 50), multifunctional ABC-family protein with diverse, essential roles in protein synthesis (48). Therefore, Rli1 is also present in Plasmodium spp. Sequence identity with the yeast Rli1 protein is 59% in the human pathogen P. falciparum (the PF3D7_1368200 gene). As indicated above, Rli1 function has been shown to be a primary cellular target of ROS and redox-active agents such as H_2_O_2_, paraquat, copper (34), and primaquine (18). Decreased Rli1 activity is known to result in stop codon readthrough (37), similar to the effect of MMV665909. The N-terminal [4Fe-4S] cluster domain of Rli1 plays a crucial role in its functions (including accurate stop codon reading), while FeS clusters are known to be ROS-hypersensitive structures. Analysis of incorporation and turnover of radiolabeled ^55^Fe to Rli1 under copper stress established that FeS cluster supply to Rli1 was the primary target (34), indicating impairment by a stressor at upstream steps in FeS cluster biogenesis. The FeS cluster biogenesis process is well conserved through evolution (51, 52). In Plasmodium, three pathways are involved in FeS cluster biogenesis: the SUF (SUlFur mobilization) pathway in the apicoplast organelle, the iron-sulfur cluster (ISC) formation pathway in the mitochondrion, and the cytosolic iron-sulfur protein assembly (CIA) pathway, which resides in the cytosol and nucleus. The ISC/CIA pathways are essential in the maturation of Rli1 and common to yeast and Plasmodium spp. Therefore, an Rli1-targeted mechanism of MMV665909 action, as suggested here, is likely to be well conserved.

In a previous study, MMV665909 was shown to inhibit the interaction between proteins PfAtg8 and PfAtg3 (53). Atg8 is a ubiquitin-like autophagy protein, and Atg3 is its E2-conjugating enzyme. Atg8 is essential for Plasmodium growth and survival and partially localizes to the apicoplast. Yeast expresses an Atg8 orthologue; but the protein is not essential in yeast, and therefore any inhibition by MMV665909 could not alone account for inhibition of cell growth. Autophagy and translation are linked processes. Ribophagy is an autophagic pathway that targets ribosomes (54). Work in P. falciparum showed that PfAtg8 may possibly be involved in ribophagy (55). Any MMV665909-mediated impairment of ribophagy via PfAtg8 would abrogate normal control of protein synthesis, thus potentially exacerbating the effects of error-prone translation caused by any depletion of functional Rli1 by the same drug. In addition, bioinformatic predictions at ChEMBL (www.ebi.ac.uk/chembl/) suggest that the lysine and proline tRNA synthetases may be targets of MMV665909. It is possible that any targeting of these translation-related enzymes has the potential to contribute further to mistranslation. It is not unexpected or rare for a single drug to have multiple targets. For example, the major antimalarial artemisinin targets both mitochondria (15) and the calcium channels Pmr1 and Pmc1 (56) in yeast.

To help tackle concerns over the development of resistance, antimalarials are now commonly administered as combination therapies. This strategy is known to improve efficacy of treatment and reduce the risk of resistance emergence. Moreover, drugs targeting protein synthesis, like the recently identified DDD107498 (3), need to be combined with a fast-acting compound that reduces the initial level of infection. A similar strategy would probably apply to MMV665909, given the action on fidelity of protein synthesis described here. The current antimalarials amodiaquine, chloroquine, and primaquine are all reported to promote oxidative stress (18, 57–59) in common, we argue, with MMV665909 (Fig. 2 and 3). ROS-labile FeS groups have been described as the primary targets of PMQ (18). An increase of oxidized proteins was observed in parasites treated with CQ (58). CQ-heme complexes in the parasite may generate oxidative stress by enhancing the toxicity of the ROS produced during the degradation of the hemoglobin (57). Furthermore, quinoline antimalarials can deplete certain essential amino acids like tryptophan and tyrosine (16, 23), an effect likely to decrease translation fidelity (40, 41). Therefore, we tested quinoline derivatives in combination with MMV665909, and we observed marked synergy. Synergistic combinations allow lower doses of the drugs to be used than if the drugs are supplied singly, which lessens cost and risk of toxicity. Toxicity is a particular concern for drugs like primaquine, which is associated with severe side effects and causes hemolysis in patients with glucose-6-phosphate dehydrogenase deficiency (60).

This study has exploited the power of yeast genetic tools to show that the candidate antimalarial MMV665909 is able to target the fidelity of protein translation, probably via the essential FeS protein Rli1, revealing a novel mode of action for an antimalarial. Rli1 is highly conserved, including in Plasmodium spp. Furthermore, MMV665909 was shown to act in synergy with the current antimalarials chloroquine, amodiaquine, and primaquine. Therefore, this study supports translation fidelity as a novel target for antimalarials such as MMV665909, a candidate MMV drug in the fight against malaria.

## MATERIALS AND METHODS

### Yeast strains and plasmids.

Unless specified otherwise, all experiments were performed with Saccharomyces cerevisiae BY4741 (*MAT***a** *his3-1 leu2-0 met15-0 ura3-0*). Isogenic deletion mutants were from Euroscarf (Frankfurt, Germany). The double deletion mutant *sul1Δ sul2Δ* was constructed previously (33). The S. cerevisiae W303 background (*MAT*α *ura3-1* *ade2-1* *trp1-1* *his3-11*,*15 leu2-3*,*112*) was used for red/white mistranslation assays. Yeast were maintained and grown in YPD medium (2% peptone [Oxoid, Basingstoke, United Kingdom], 1% yeast extract [Oxoid], 2% d-glucose) or YNB medium (0.69% yeast nitrogen base without amino acids; Formedium, Norfolk, United Kingdom) supplemented with 2% (wt/vol) d-glucose and as appropriate for plasmid selection (61). Where necessary, medium was solidified with 2% (wt/vol) agar (Sigma-Aldrich, St. Louis, MO). For overexpression of proteins, the *RLI1* or *SUP45* open reading frames (ORFs) were placed under the control of the *tetO* promoter in the pCM190 vector and modified so that the product was C-terminally tagged with the hemagglutinin (HA) epitope, as described previously for pCM190-*RLI1-HA* (34). *SUP45* was ligated between the NotI-PstI sites of pCM190. Yeast transformations were performed by the lithium acetate method (62).

### Chemicals.

With the exception of the compounds from the MMV box provided by the Medicines for Malaria Venture (Geneva, Switzerland), all drugs were from Sigma-Aldrich: paromomycin sulfate, amodiaquine dihydrochloride dihydrate, chloroquine diphosphate salt, and primaquine bisphosphate. With the exception of MMV compounds (in dimethyl sulfoxide [DMSO]), stock solutions of all chemicals used in this study were prepared in distilled water, filter sterilized, and added to growth medium to give the final concentrations specified in the figure legends or on the figures.

### Growth inhibition assays.

Single colonies of yeast were used to inoculate broth cultures in Erlenmeyer flasks and incubated at 30°C with orbital shaking at 120 rpm overnight. Overnight cultures were diluted to an OD at 600 nm (OD_600_) of ∼0.5 and cultured for a further 4 h in fresh medium. The 4-h mid-/late-exponential-phase cultures were diluted to an OD_600_ of ∼0.1, and 300-μl aliquots were transferred to 48-well microtiter plates (Greiner Bio-One, Stonehouse, United Kingdom) with chemicals added as specified in the figure legends or on the figures and balanced for any solvent additions. Plates were incubated at 30°C with shaking in a BioTek Powerwave XS microplate spectrophotometer, and the OD_600_ was recorded every 30 min.

### Checkerboard assays.

All culturing for checkerboard assays was performed as described above. Aliquots (150 μl) were transferred to 96-well microtiter plates (Greiner Bio-One, Stonehouse, United Kingdom) with chemicals added as specified on Fig. 4B. The inoculated plates were incubated statically for 24 h at 30°C before measurement of the OD_600_ with a BioTek EL800 microplate spectrophotometer. After subtraction of the background reading for noninoculated medium, growth for each condition was calculated as a percentage of the growth of the control in the absence of the added inhibitors. Fractional inhibitory concentrations (FICs) were calculated as described previously (63).

### Anaerobic growth assays on solid medium.

S. cerevisiae W303 cultures prepared as described above were adjusted to OD_600_ values of ∼2.0, 0.2, 0.02, and 0.002, and the dilution series was spotted (4 μl) onto YPD agar alone or supplemented with the MMV drug. Images were captured after 4 days of growth at 30°C under anaerobic (Whitley DG250 anaerobic workstation; Don Whitley Scientific) or aerobic conditions.

### Mistranslation assays.

For qualitative determination of mistranslation, experimental cultures of S. cerevisiae W303 were spotted onto YPD agar alone or supplemented with the MMV drug, as described above. Images were captured for comparisons of red versus white colonies after 2 days of growth at 30°C.

For quantitative determination of mistranslation, S. cerevisiae was transformed with a dual-luciferase reporter plasmid encoding firefly and Renilla luciferases either separated by a stop codon (UAA version kindly provided by D. Bedwell, University of Alabama [47], or UGA version [in a *BSC4* context] supplied by C. Loenarz, University of Nottingham [64]) or containing a missense codon in the ORF encoding firefly luciferase (His245 → Arg245; pDB868 from D. Bedwell [46]). Precultures were prepared as described above in YNB broth supplemented appropriately for plasmid selection. Then, the cultures were diluted to an OD_600_ of ∼0.1 in YPD medium, 300-μl aliquots were transferred to 48-well microtiter plates, and the MMV drug was added as specified in the figure legends or on the figures. Plates were incubated at 30°C for 16 h with shaking in a BioTek Powerwave XS microplate spectrophotometer or statically in the presence or absence of oxygen for the anaerobic or aerobic assays, respectively. Cell extracts were prepared by lysis of culture samples (OD_600_ of ∼2) for 10 min using passive lysis buffer from a Promega Dual-Luciferase Reporter Assay System (Promega, Madison, WI, USA). Firefly luciferase activity was measured (10-s integration time) using luciferase assay buffer (Promega) in a GloMax 20/20 luminometer (Promega). Renilla luciferase activity (10-s integration time) was determined subsequent to quenching of firefly activity using Stop & Glo buffer (Promega). Background measurements for nontransformed cells were subtracted, and the ratio of luminescence attributable to the firefly versus Renilla luciferase was calculated.

## Supplementary Material

Supplemental material
