# Supplementary material for: The Candidate Antimalarial Drug MMV665909 Causes Oxygen-Dependent mRNA Mistranslation and Synergizes with Quinoline-Derived Antimalarials
Source: Antimicrob Agents Chemother. 2017 Aug 24;61(9):e00459-17. doi: 10.1128/AAC.00459-17 (PMC5571370; doi:10.1128/AAC.00459-17)
Supplement: Supplemental material [file supp_61_9_e00459-17__index.html]

Supplemental material 

# The Candidate Antimalarial Drug MMV665909 Causes Oxygen-Dependent mRNA Mistranslation and Synergizes with Quinoline-Derived Antimalarials

## Supplemental material

- Supplemental file 1 -

  Fig. S1-S3

  PDF, 420K
