# Supplementary material for: The Candidate Antimalarial Drug MMV665909 Causes Oxygen-Dependent mRNA Mistranslation and Synergizes with Quinoline-Derived Antimalarials
Source: Antimicrob Agents Chemother. 2017 Aug 24;61(9):e00459-17. doi: 10.1128/AAC.00459-17 (PMC5571370; doi:10.1128/AAC.00459-17)
Supplement: Supplemental material [file AAC.00459-17_zac009176488s1.pdf]

SUPPLEMENTAL MATERIAL

Vallières and Avery

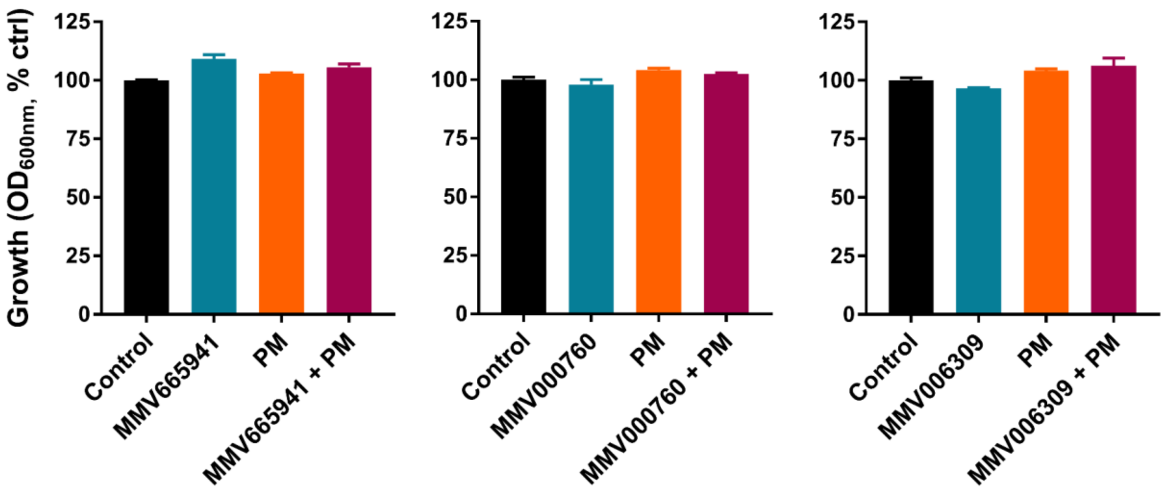

FIG S1 MMV drugs that did not act synergistically with paromomycin. Growth of *S. cerevisiae* in YPD broth supplemented or not with just sub-inhibitory concentrations of 1 µM MMV665941, 10 µM MMV000760, 100 µM MMV006309, and/or 200 µg.ml^{-1} paromomycin (PM). OD_{600} was measured after 15 h. Percentage growth is calculated relative to control growth minus drug. Mean data are shown from duplicate independent experiments ± SEM.

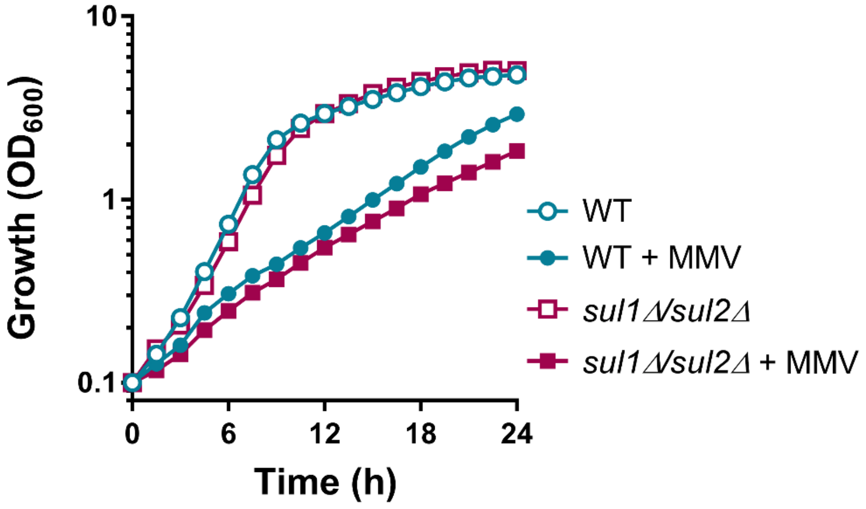

FIG S2 Deletion of sulphate transporters does not rescue the growth inhibitory action of MMV665909. *S. cerevisiae* BY4741 and isogenic deletion mutant *sul1Δ/sul2Δ were* incubated in YPD broth supplemented or not with 30 µM MMV665909. SEMs from triplicate independent growth experiments are smaller than the dimensions of the symbols.

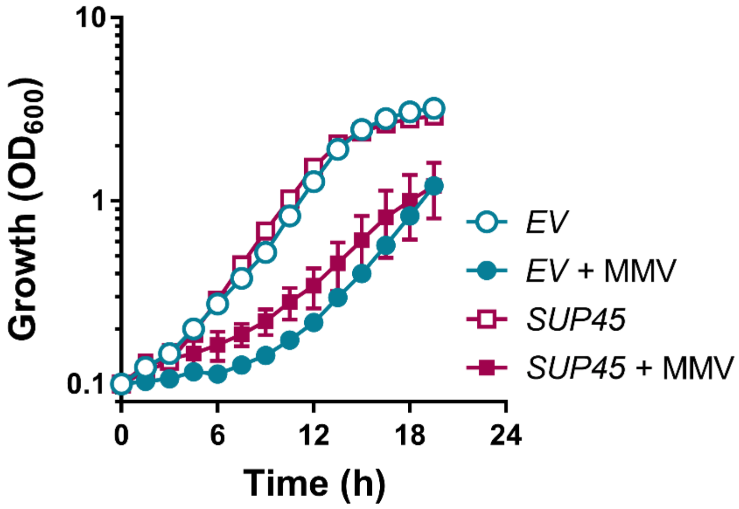

FIG S3 Overexpression of *SUP45* does not rescue yeast treated with MMV665909. *S. cerevisiae* transformed with the *tet* bearing plasmid, either empty (EV) or overexpressing *SUP45*, were cultured in YNB medium supplemented or not with 10 µM MMV665909. Doxycycline was excluded to give maximal *SUP45* expression. SEMs from triplicate independent growth experiments are smaller than the dimensions of the symbols.
